# A GLP-1 analogue optimized for cAMP-biased signaling improves weight loss in obese mice

**DOI:** 10.1016/j.molmet.2025.102124

**Published:** 2025-03-27

**Authors:** Jonathan D. Douros, Aaron Novikoff, Barent DuBois, Rebecca Rohlfs, Jacek Mokrosinski, Wouter F.J. Hogendorf, Robert Augustin, Myrte Merkestein, Lene Brandt Egaa Martini, Lars Linderoth, Elliot Gerrard, Janos Tibor Kodra, Jenny Norlin, Nikolaj Kulahin Roed, Anouk Oldenburger, Stephanie A. Mowery, Maria Waldhoer, Diego Perez-Tilve, Brian Finan, Steffen Reedtz-Runge, Timo D. Müller, Patrick J. Knerr

**Affiliations:** 1 Novo Nordisk Research Centre Indianapolis, Indianapolis, IN, USA; 2 Institute for Diabetes and Obesity, Helmholtz Munich, Neuherberg, Germany; 3 German Center for Diabetes Research (DZD), Neuherberg, Germany; 4 Novo Nordisk A/S, Måløv, Denmark; 5 Department of Pharmacology and Systems Physiology, University of Cincinnati College of Medicine, Cincinnati, OH, USA; 6 Walther-Straub-Institute for Pharmacology and Toxicology, Ludwig-Maximilians-University Munich, Munich, Germany

**Keywords:** GLP-1, Biased agonism, Semaglutide, Obesity

## Abstract

**Objective:**

Glucagon-like peptide 1 (GLP-1) receptor (GLP-1R) agonism is foundational to modern obesity pharmacotherapies. These compounds were engineered for maximal G protein alpha(s) (Gsα) signaling potency and downstream cAMP production. However, this strategy requires reconsideration as partial, biased GLP-1R agonists characterized by decreased Gsα signaling and disproportionate reductions in β-arrestin recruitment relative to the native ligand provide greater weight loss than full, balanced agonists in preclinical models.

**Methods:**

We tested the hypothesis that *in vitro* signaling bias, which considers both cAMP signaling and β-arrestin recruitment, better predicts weight loss efficacy in diet induced obese (DIO) rodents than cAMP potency alone.

**Results:**

Our data demonstrate that signaling bias significantly correlates to GLP-1R agonist mediated weight loss in diet-induced obese mice. We further characterized a protracted GLP-1 analogue (NNC5840) which exhibits a partial-Gsα, cAMP-biased GLP-1R signaling profile *in vitro* and demonstrates superior maximal body weight reduction compared to semaglutide in DIO mice. The NNC5840 weight loss profile is characterized by reduced *in vivo* potency but increased maximal efficacy.

**Conclusion:**

The data demonstrate that biased agonism is a strong predictor of *in vivo* efficacy for GLP-1R agonists independent of factors like intrinsic cAMP potency or pharmacokinetics. These data suggest that drug discovery screening strategies which take a holistic approach to target receptor signaling may provide more efficacious candidate molecules. The interpretations of these studies are limited by unknowns including how structural modifications to the biased GLP-1R agonist effect physiochemical properties of the molecules.

## Highlights

• *In vitro* cAMP signaling bias at the GLP-1R correlates to *in vivo* weight loss in DIO mice.
• NNC5840 exhibits a partial-Gsα, cAMP-biased GLP-1R signaling profile *in vitro.*
• NNC5840 demonstrates greater maximal weight loss than semaglutide in DIO mice.

## 1 Introduction

Modern obesity therapy is reliant on drugs that activate the GLP-1R, including semaglutide and tirzepatide. GLP-1R activation drives Gsα recruitment and downstream cyclic adenosine monophosphate (cAMP) production; cAMP is recognized as a primary driver for GLP-1R action. Subsequent β-arrestin recruitment to the GLP-1R is classically associated with receptor internalization and signal desensitization [1]. During the discovery process of long-acting GLP-1R agonists, notably semaglutide, the molecular engineering and *in vitro* pharmacology primarily focused on optimizing Gsα/cAMP signaling potency and prolonged half-life, with little consideration of β-arrestin recruitment, under the assumption that this would result in maximal efficacy *in vivo* [2,3]. However, recent studies call this assumption into question. Several compounds, including the GIPR:GLP-1R co-agonists tirzepatide and CT-859, are reported to exert partial and biased Gsα signaling at the GLP-1R (reviewed in [4]) [[5], [6], [7], [8]]. These molecules are characterized by reduced Gsα signaling/cAMP production potency (i.e. EC_50_) and efficacy (i.e. E_max_) *in vitro* relative to native GLP-1, along with disproportionate decreases in β-arrestin recruitment. This results in a positive cAMP:β-arrestin signaling ratio (i.e. cAMP-biased) relative to native GLP-1, which is by definition balanced [4]*.* Despite the partial Gsα signaling profile, these drugs counterintuitively induce greater weight reduction [[5], [6], [7], [8], [9]] and insulinotropic [9] efficacy in rodents *in vivo* compared to high-potency, full-efficacy, balanced agonists.

Based on these data, we hypothesized that the ligand mediated *in vitro* signaling bias metric β, calculated as the ratio of *in vitro* cAMP:β-arrestin signaling (E_max_ x pEC_50_) for a given test compound (e.g. NNC5840) relative to a reference molecule (e.g. native GLP-1) [10], is a stronger predictor of preclinical *in vivo* weight loss than cAMP potency alone. By analyzing a small panel of GLP-1R agonists, we demonstrate that β, but not cAMP signaling alone, significantly correlates to *in vivo* weight loss in DIO mice. This finding is reinforced by our characterization of a fatty-acylated GLP-1 analogue, NNC5840, whose partial Gsα, cAMP-biased signaling profile drives superior maximal weight lowering than the full, balanced agonist semaglutide in rodents. The findings suggest a need to expand the canonical model of GLP-1R pharmacology, and likely other GPCRs, to incorporate *in vitro* biased agonism as a determinant of *in vivo* efficacy. This revamped model will not only improve our fundamental understanding of receptor biology but also guide future drug discovery efforts.

## 2 Methods

### 2.1 Peptide synthesis

All peptides were generated using standard fluorenylmethoxycarbonyl-based solid-phase synthesis and purified by reversed-phase high-performance liquid chromatography as has been reported previously [11].

### 2.2 *In vitro* assays

**Figure 1:**
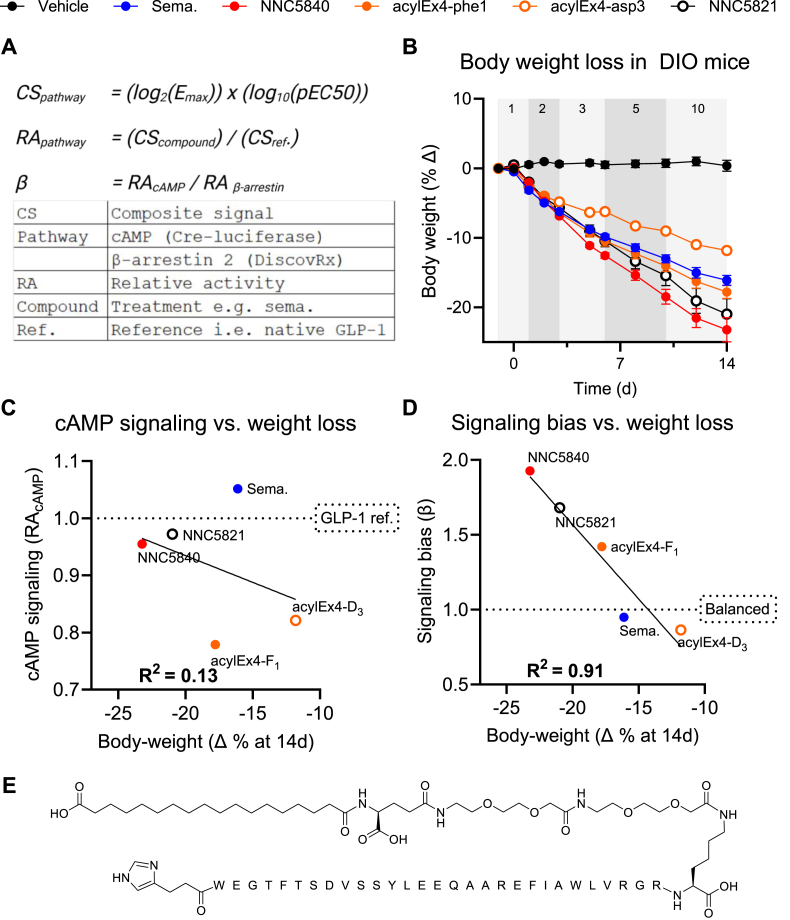
**Signaling bias better predicts body-weight loss by GLP-1R agonists in mice than cAMP signaling**. (A) Calculation of the signaling bias factor β. (B) Body-weight reduction for five GLP-1R agonists over 14 days. The doses (nmol/kg) used on each day are reported at the top of the graph. Relationship between body-weight loss shown in panel B with (C) cAMP signaling as quantified by RA_cAMP_ or (D) β. (E) Structure of NNC5840.

The CRE-Luciferase reporter assay used to assess cAMP production in Figure 1 and Supplemental Table 1 has been reported previously [[11], [12], [13]]. Briefly, stably transfected baby hamster kidney (BHK) cell lines expressing GLP-1R and firefly luciferase reporter gene linked to the cAMP response element (CRE) were seeded in poly-d-lysine-coated 96 well opaque well tissue culture plates at 5,000 cells per well in growth media, incubated overnight, and washed once in Dulbecco's phosphase-buffered saline (DPBS). To each well was added 50 μL of assay buffer (DMEM without phenol red, 10 mM HEPES, 1 × Glutamax, 1% ovalbumin, 0.1% Pluronic F-68) containing serial dilutions of test compounds. The test plates were incubated at 37 °C for 3 h in a CO_2_ incubator, then washed once with 100 uL per well of DPBS followed by addition of 100 μL per well of SteadyLite plus reagent (PerkinElmer). The assay plates were covered to protect reagent from light, shaken at 250 rpm at room temperature for 30 min, and read in a microtiter plate reader. EC_50_ values were calculated using Prism software (GraphPad) as the nonlinear regression of log(compound concentration) vs. response.

**Figure 2:**
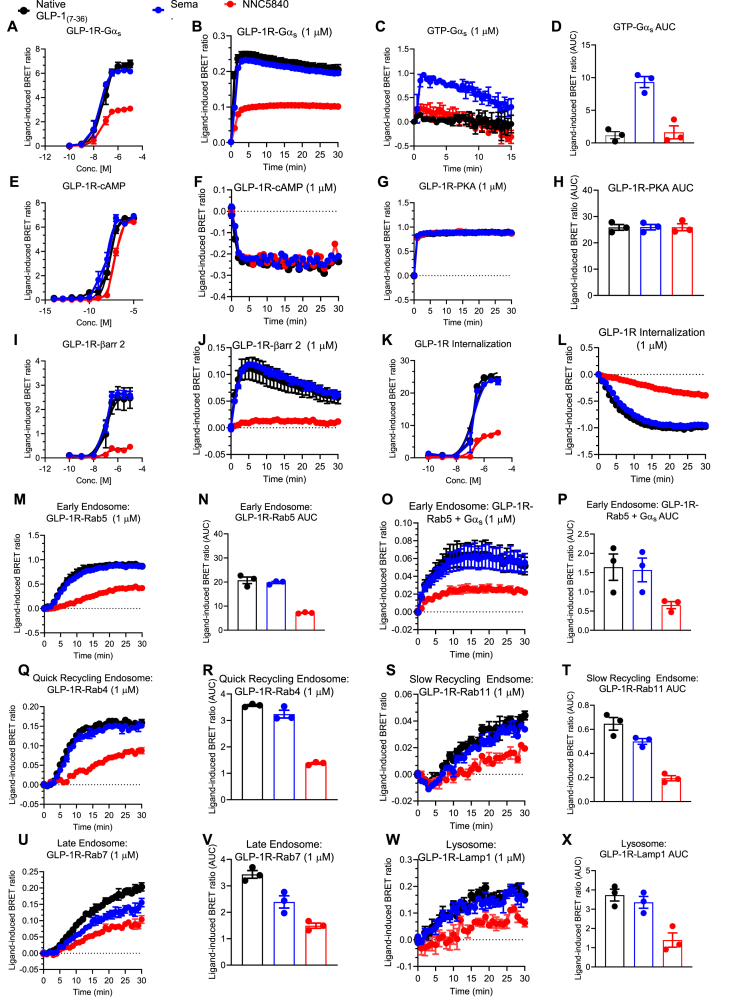
**NNC5840 displays partial agonism that is biased toward Gs compared to semaglutide**. The effect of native GLP-1, semaglutide, and NNC5840 on GLP-1R mediated (A,B) miniGsα recruitment; (C,D) endogenous GTP production; (E,F) cAMP production; (G,H) PKA activation; (I,J) β-arrestin 2 recruitment; and (K,L) receptor internalization *in vitro*. The effect of native GLP-1, semaglutide, and NNC5840 on (M,N) GLP-1R co-localization into Rab5^+^ endosomes; and (O,P) signaling by Rab5^+^ Gsα *in vitro.* GLP-1R co-localization into (Q, R) Rab4^+^, (S,T) Rab11^+^, and (U,V) Rab7^+^ endosomes, and (W,X) LAMP1^+^ lysosomes *in vitro.*

The *in vitro* assays reported in Figure 2 have been reported previously [14]. Briefly, HEK293T cells (ATCC) were cultured in Dulbecco's Modified Eagle Medium (DMEM) supplemented with 10% heat-inactivated fetal bovine serum (FBS), 100 IU/mL of penicillin, and 100 mg/mL of streptomycin solution. HEK293T cells (700,000/well) were seeded in 6-well plates and incubated to 70% confluency in DMEM (10% FBS, 1% Pen/Strep) and incubated for 24 h. Transient transfections were then performed using Lipofectamine 2000 according to the manufacturer's protocol and then incubated for 24 h. After transfection, the cells were washed with PBS, detached, and resuspended in FluoroBrite phenol red-free complete media (Cat #: A1896701, Life Technologies, Carlsbad, CA, USA) containing 5% FBS and 2 mM of l-glutamine (Cat #: 25030081, Life Technologies, Carlsbad, CA, USA). Then 100,000 cells/well were plated into poly-d-lysine-coated (Cat #: P6403, Sigma–Aldrich, St. Louis, MO, USA) 96-well white polystyrene LumiNunc microplates (Cat #: 10072151, Thermo Fisher Scientific, Waltham, MA, USA). After 24 h, the media was replaced with PBS (Cat #: 10010056, Gibco, Carlsbad, CA, USA) containing 10 μM of coelenterazine-h (Cat #: S2011, Promega, Madison, WI, USA) or 1:500 NanoGlo (Cat #: N1110, Promega, Madison, WI, USA). BRET measurements were taken every 60 s using a PHERAstar FS multi-mode microplate reader. Ligand-induced dynamics in GLP-1R signaling or trafficking were measured as subsequent changes relative to baseline (after time point zero). Each experiment was independently performed at least three times, with at least three technical replicates for each group. Positive or negative incremental areas under the curve (iAUC) were calculated and represented for dose–response relationships. Either hGLP-1R untagged (Sino Biological Inc.), hGLP-1R-GFP (a kind gift from D. Hodson; University of Oxford, Oxford, England), or hGLP-1R-Rluc8 (a kind gift from P. Sexton; Monash University, Melbourne, Australia) were utilized within various combinations of MiniGαs/MiniGαq (a kind gift from N. Lambert; Augusta University, Augusta, GA, USA), indirect GTP-bound Gα sensor Gβγ-BERKY3 (a kind gift from M. Garcia-Marcos; Addgene plasmid # 158219), cAMP-sensor pcDNA3L-His-CAMYEL (Cat# MBA-277, ATCC), plasma membrane marker EGFP-CAAX (a kind gift from L. Lu; Addgene plasmid # 86056), PKA activity-sensor ExRai-AKAR2 (a kind gift from J. Zhang; Addgene plasmid # 161753), endosomal markers mEmerald-Rab5, mEmerald-Rab4, mEmerald-Rab11, mEmerald-Rab7 (kind gifts from M. Davidson), and the lysosomal marker mNeonGreen-Lamp1 (a kind gift from D. Gadella; Addgene plasmid # 98882).

We calculated the signaling bias metric β for each test compound (compound) relative to the reference compound native GLP-1 (ref) as follows:

*Composite signal (CS) for a given test compound at the cAMP and β-arrestin pathway:*

*CS*_*pathway*_*= (log*_*2*_*(E*_*max*_*)) x (log*_*10*_*(pEC50))*

*Relative activity (RA) for a given test compound at the cAMP and β-arrestin pathway:*

*RA*_*pathway*_*= (CS*_*compound*_*) / (CS*_*ref*_*.)*

*The signaling bias metric β:*

(1) $$\beta ={\mathrm{RA}}_{\mathrm{cAMP}}/{\mathrm{RA}}_{\mathrm{\beta -arrestin}}$$

### 2.3 Weight loss studies

All animal studies were performed at the University of Cincinnati in accordance with approved IACUC protocols. DIO mice were given *ab libitum* access to water and a 58% fat, high-sugar diet (D12331, Research Diets) for at least 12 weeks and housed 3–4 per cage. Mice were exposed to a controlled 12 h/12 h light–dark cycle at room temperature (22 °C). Male C57B6/J or MS-NASH mice (Jackson Labs) were randomized and evenly distributed to test groups (n = 8 per group) according to body weight at < 9 months of age. GLP-1R agonist treatment began on day 0 for each study. Treatments were administered via daily subcutaneous (SC) injection for the duration and dosage indicated. Dose escalation regimens for each peptide were determined based on previously published studies [11]. Body weight and food intake were measured every other day throughout the study.

### 2.4 Pharmacokinetics

For pharmacokinetic (PK) studies, wild-type mice were dosed SC with semaglutide or NNC5840 as described above. Due to blood volume collection restrictions, we followed a standardized sparse sampling procedure in which mice from each group were randomized into two subgroups (n = 4/subgroup) that were sampled at alternating time points over 24 h. Thus, the PK profiles are an overall average of 8 mice/group and 4 mice/subgroup sampled at each time point. PK profiles were assessed according to previously reported methods [15]. Briefly, plasma concentration–time profiles were analyzed by a non-compartmental method (Pharsight Phoenix WinNonLin v.6.4). The terminal half-life (t_1/2_), maximum plasma concentration (C_max_), time for maximum plasma concentration (t_max_), and AUC from zero to last (AUC_0-t_) were determined. Criteria for estimation of t_1/2_ were at least three concentration–time points in the terminal phase not including Cmax, with an R^2^ ≥ 0.85.

### 2.5 LC/MS bioanalysis

Plasma concentrations of NNC5840 and semaglutide were determined by liquid chromatography-tandem mass spectrometry (LC-MS/MS) using a multiple reaction monitoring method. Briefly, plasma proteins were precipitated by mixing plasma samples with 6 volumes of methanol-containing internal standard in micronic 1.5 mL microcentrifuge tubes*,* followed by centrifugation for 20 min at 13,000×*g*. The supernatant was transferred to a 96-well plate and diluted with water containing 0.1% formic acid and mixed thoroughly. Diluted samples were injected into the LC-MS/MS system. The chromatographic separation was performed on a Thermo Scientific Vanquish LC system using a Waters Acquity UPLC BEH C18 column (1.0 mm × 50 mm, 1.7 μm) with gradient elution of 0.1% formic acid in water (mobile phase A) and 0.1% formic acid in acetonitrile (mobile phase B) at a flow rate of 0.3 mL/min with a column temperature of 60 °C. The mass spectrometric detection was performed on a Thermo Scientific TSQ Quantis triple quadrupole system with electrospray ionization in positive ion mode.

### 2.6 Statistics

*The in vivo* weight loss studies were assessed using a 2-way ANOVA with Tukey's posthoc correction in GraphPad Prism.

## 3 Results

### 3.1 Biased signaling metric β is significantly correlated with *in vivo* weight loss

We tested the hypothesis that the signaling bias metric β (Fig. 1A) [10,16] is superior to cAMP potency for predicting the preclinical *in vivo* weight lowering efficacy of GLP-1R agonists. The *in vitro* signaling profile including cAMP generation and β-arrestin recruitment was assessed for five GLP-1R agonists, using native GLP-1 as the reference compound: the balanced agonists semaglutide and acylEx4-asp3 [5]; previously reported biased agonist acylEx4-phe1 [5]; and novel fatty-acylated biased GLP-1R agonists NNC5840 and NNC5821 (Supplemental Table 1). Each GLP-1R agonist was administered via daily SC injection in DIO C57B6/J mice over 14 days (range: −11.81% to −23.22 %; Figure 1B). cAMP accumulation (RA_cAMP_), as assessed using the CRE-Luciferase reporter assay used as a proxy for Gsα signaling, did not show a significant correlation to weight loss (R^2^ = 0.13, deviation from 0 p-value = 0.47; Figure 1C). Conversely, β showed a significant correlation with weight loss (R^2^ = 0.91, deviation from 0 p-value = 0.01; Figure 1D), indicating that signaling bias is a stronger predictor of weight reducing efficacy *in vivo* than cAMP signaling*.* However, this conclusion is limited by the lack of pharmacokinetic data for all molecules used and weight-loss not yet reaching E_max_. Therefore, we selected a partial, biased GLP-1R agonist (NNC5840, Fig. 1E) for further assessment of its PK/pharmacodynamic (PD) weight loss profile compared to the balanced, full agonist semaglutide.

### 3.2 NNC5840 is a partial-Gsα, cAMP-biased GLP-1R agonist

First, we performed an *in vitro* characterization of NNC5840 compared to semaglutide in GLP-1R^+^ HEK293T cells and BRET-based reporter assays to assess GLP-1R signaling, internalization, and endosomal and lysosomal trafficking. NNC5840 partially agonizes GLP-1R G protein signaling compared to native GLP-1_(7-36)_ and semaglutide, as measured by synthetic miniGsα and miniGqα recruitment in dose–response and temporal measurements (Figure 2A,B; Supplemental Fig. 1). Similarly, NNC5840 is a partial activator of endogenous GTP production (Figure 2C,D). Despite this, NNC5840 retains maximal cAMP production and PKA activation relative to GLP-1_(7-36)_ and semaglutide, albeit with ∼10x reduced cAMP potency assessed via BRET-based assay (EC_50_ for NNC5840 = 73.9 nM, semaglutide = 7.6 nM; Figure 2E–H). NNC5840 exhibits significantly lower GLP-1R β-arrestin 2 recruitment and GLP-1R internalization relative to GLP-1_(7-36)_ and semaglutide (Figure 2I-L). Consequentially, NNC5840 stimulates less GLP-1R co-localization into Rab5^+^ early endosomes and subsequent signaling by Rab5^+^ Gsα (Figure 2M−P). GLP-1R localization with Rab4^+^ ‘quick’ recycling endosomes and Rab11^+^ ‘slow’ recycling endosomes is reduced after NNC5840 treatment relative to the comparators (Figure 2Q-T). Lastly, Rab7^+^ late endosome and LAMP1^+^ lysosome co-localization is diminished after NNC5840 relative to both controls, suggesting NNC5840 limits ligand mediated receptor degradation (Figure 2U–X). Broadly, NNC5840 demonstrates a partial-Gsα, cAMP-biased signaling profile along with altered trafficking characteristics *in vitro*. The pharmacologic profile elicited by NNC5840 predisposes the GLP-1R to minimal internalization and, likely, reduced signal desensitization.

### 3.3 NNC5840 elicits greater maximal weight loss than semaglutide in DIO rodents

**Figure 3:**
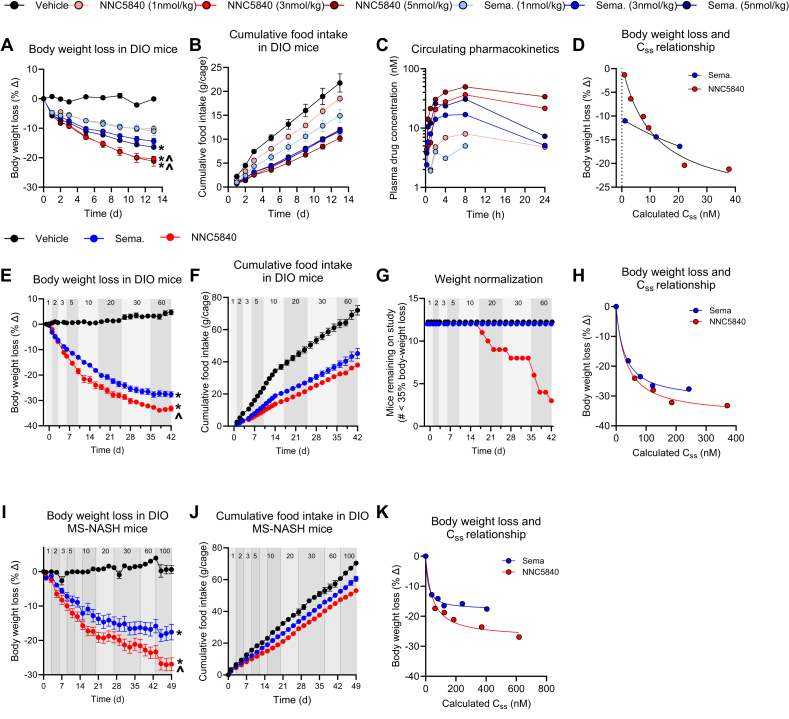
**NNC5840 induces greater maximal body weight loss than semaglutide in mice.** (A) Body weight loss, (B) cumulative food intake, and (C) circulating compound concentrations in DIO mice measured after the first injection (d0) with fixed doses of either semaglutide or NNC5840 (1, 3, 5 nmol/kg). (D) Body weight loss at day 13 (data curated from Figure 3A and Supplemental Fig. 2), as a function of the calculated circulating drug exposure in the study shown in panels A–C and Supplementary Table 2. (E) Body weight loss, (F) cumulative food intake, and (G) number of animals remaining on study (animals are removed at 35% body weight loss) in DIO mice given semaglutide or NNC5840 at doses escalating from 1 to 60 nmol/kg as indicated at the top of each graph. (H) Body weight loss at time of last dosing plotted against calculated circulating drug exposure for doses of 10–60 nmol/kg in the study shown in panels E–G. (I) Body weight loss and (J) cumulative food intake in DIO MS-NASH mice given semaglutide or NNC5840 at doses escalating from 1 to 100 nmol/kg as indicated at the top of each graph. (K) Body weight loss at time of last dosing plotted against calculated circulating drug exposure for doses of 10–100 nmol/kg in the study shown in panels I–J. ∗ represents p-value <0.05 compared to vehicle; ˆ represents p-value <0.05 between NNC5840 and semaglutide (for panel A, at the same dose).

We advanced NNC5840 to *in vivo* dose response studies comparing its effects to semaglutide in male C57B6/J DIO mice. NNC5840, semaglutide, and the semaglutide surrogate NNC2220, which exhibits comparable chemical and *in vitro/in vivo* pharmacologic properties to semaglutide [17], elicit dose-dependent reductions in body weight and food intake (Figure 3A,B; Supplemental Fig. 2). The magnitude of weight loss and food intake reduction induced by NNC5840 is similar to semaglutide and NNC2220 at doses between 0.3 and 1.5 nmol/kg (Figure 3A, Supplemental Fig. 2). However, NNC5840 induces greater weight loss than that of semaglutide at higher doses (3–5 nmol/kg; Figure 3A). The PK profile of NNC5840 in DIO mice exhibits greater exposure over a 24h time course compared to semaglutide (Figure 3C; Supplemental Table 2), suggesting the superior maximal weight loss could simply be due to increased exposure. However, the PK/PD relationship combining weight loss data from Figure 3A and Supplemental Fig. 2 suggests the potential that NNC5840 may be more efficacious than semaglutide with respect to maximal body weight loss (E_max_ −28.76% for NNC5840 vs. −17.82% for semaglutide; Figure 3D), albeit with less potency (ED_50_ = 11.13 nmol/kg for NNC5840 vs 2.03 nmol/kg for semaglutide).

Because these PK/PD data suggest, but do not conclusively demonstrate, that NNC5840 induces superior weight loss at equivalent circulating drug exposures, we performed a dose escalation study in DIO mice comparing NNC5840 and semaglutide. We again show that semaglutide induces statistically similar weight loss at low doses (1–2 nmol/kg) and calculated C_ss_ relative to NNC5840 (Figure 3E). However, at higher doses (5–60 nmol/kg), NNC5840 induces greater weight loss relative to semaglutide. Additionally, NNC5840 appears to elicit greater maximal weight loss than semaglutide. Semaglutide is maximally efficacious at 30 nmol/kg, in keeping with previous results. Conversely, the apparent plateau in weight loss induced by NNC5840 is an experimental artifact, as animals must be removed from the study at >35% weight loss per humane use of animal protocols. Further analysis reveals that no animals given semaglutide achieve >35% weight loss at doses up to 60 nmol/kg, while mice given NNC5840 achieve >35% weight loss at doses as low as 20 nmol/kg (n = 3; Figure 3G). Additionally, five mice reach the 35% weight loss mark when escalating NNC5840 doses from 30 to 60 nmol/kg, further suggesting an increase in the maximally efficacious dose for NNC5840 relative to semaglutide. The PK/PD relationship in this dose escalation paradigm plots the weight loss at the end of the dosing period for doses of 10–100 nmol/kg on the y-axis plotted against the calculated C_ss_ on the x-axis (Figure 3H). This relationship exhibits similar trends to those in the dose response study. NNC5840 appears less potent than semaglutide (ED_50_ 32.72 nmol/kg for NNC5840 vs. 28.17 nmol/kg for semaglutide) but more efficacious (E_max_ −36.56% for NNC5840 vs. −31.57% for semaglutide).

In a final study, we examined the maximal effect of NNC5840 and semaglutide in MS-NASH mice [18]. These animals exhibit a dampened response to GLP-1-induced weight loss relative to DIO C57B6/J mice, which is reminiscent of the reduced weight lowering efficacy of GLP-1 drugs seen in patients with obesity and type 2 diabetes (T2D) compared to obesity alone. Importantly, because these mice are not as responsive to GLP-1R agonism, we can examine maximal weight loss at higher dose levels without animals having to be removed from the study for achieving >35% weight loss. We show that NNC5840 outperformed semaglutide, yielding greater body weight loss and food intake reduction (Figure 3I,J). A plateau in weight loss occurs for semaglutide at 30 nmol/kg. No plateau in weight loss is seen for NNC5840 even at doses up to 100 nmol/kg. In this mouse model, NNC5840 is again less potent (ED_50_ 44.25 nmol/kg for NNC5840 vs. 16.45 nmol/kg for semaglutide) but more efficacious (E_max_ −27.18% for NNC5840 vs. −17.78% for semaglutide) than semaglutide (Figure 3K). Crucially, NNC5840 more effectively reduces body weight than semaglutide at a lower calculated steady-state exposure (Css): 23% weight-loss at 375 nM Css NNC5840 vs 18% weight loss at 400 nM Css semaglutide (Figure 3K). This demonstrates that pharmacokinetic differences are not the sole determinant of the differences in efficacy between the two molecules. It should be noted that the circulating drug exposures for these maximal efficacy studies are calculated based on the data shown in Figure 3C, but not measured directly for each study. These data demonstrate that NNC5840 is more effective but less potent than semaglutide at lowering body weight. The superiority of NNC5840 cannot be solely explained by a difference in PK, and therefore could be driven primarily by the molecular pharmacology, notably cAMP bias, as suggested in Figure 1D.

## 4 Discussion

The GLP-1R is an effective pharmacologic target for treating obesity. The mechanism(s) by which drugs within the class differentiate with respect to weight-loss is unclear but has historically been attributed to the potency of a compound for generating cAMP, circulating drug exposure, and biodistribution to feeding centers of the brain. Biased GLP-1R agonism has recently emerged as another potential explanation [4], [9]. We confirm that partial-Gsα, cAMP-biased GLP-1R agonists produce more efficacious weight loss in DIO mice using a small panel of compounds. Critically, we show that signaling bias as calculated by β is a better predictor of *in vivo* weight loss efficacy than cAMP accumulation alone. β is calculated as a composite of the ratio of *in vitro* cAMP to β-arrestin signaling (E_max_ x pEC_50_) for a given test compound (e.g. NNC5840 or semaglutide) relative to a reference molecule (e.g. native GLP-1). Our data go on to characterize the novel compound NNC5840 as a partial Gsα agonist that exerts minimal β-arrestin recruitment, with an endosomal trafficking profile that preferentially maintains plasma membrane GLP-1R localization. Interestingly, despite eliciting a partial Gsα recruitment response, NNC5840 is a fully effective but less potent agonist of cAMP accumulation due to the amplification of signal between Gsα recruitment and cAMP production *in vitro*. In a variety of rodent experimental paradigms, NNC5840 exhibits a less potent but more maximally efficacious weight lowering PK/PD profile than semaglutide. Our data suggest the inclusion of biased agonism as an *in vitro* metric to help predict *in vivo* efficacy. This finding informs not only our basic biological understanding of GPCRs but also future efforts to discover maximally efficacious therapies.

GLP-1R action is generally attributed to activation of a Gsα/cAMP signaling cascade. Historically, it was assumed that more potent cAMP generation *in vitro* would yield greater efficacy *in vivo* [2,3,19]. This notion is confounded by reports that partial-Gsα, cAMP-biased GLP-1R agonists are more efficacious for glucose and weight lowering in DIO rodent models [[5], [6], [7]]. To our knowledge, all cAMP-biased agonists reported to date exhibit partial and reduced potency for Gsα recruitment with a disproportionate decrease in β-arrestin recruitment. Furthermore, it has recently been demonstrated that the GLP-1R/GIPR co-agonist tirzepatide, which exhibits a partial, cAMP-biased GLP-1R signaling profile, induced superior weight loss to semaglutide in GIPR knockout mice, thus independent of GIPR agonism [20]. Our data builds on this finding, demonstrating that *in vitro* signaling bias is a better predictor of *in vivo* efficacy than cAMP potency alone across multiple GLP-1R agonists. Furthermore, the partial-Gsα, cAMP-biased agonist NNC5840 drove greater maximal weight loss at a reduced potency in DIO rodents compared to the full, balanced agonist semaglutide, which cannot solely be explained by pharmacokinetic differences. It should be noted that our PK/PD modelling assumes dose-proportional increases in circulating drug exposure at higher doses than were empirically measured, and that the PK profile for both semaglutide and NNC5840 translate from C57B6/J to MS-NASH mice; these caveats should be considered when interpreting the data.

The exact mechanism by which signaling bias confers this paradoxical superiority is unclear. However, our data supports the hypothesis that the reduced GLP-1R internalization and degradation demonstrated *in vitro* for biased GLP-1R agonists like NNC5840 allow for the maintenance of a larger receptor pool at the plasma membrane than balanced agonists. This large, membrane localized receptor pool can be continually reengaged by agonist molecules circulating at pharmacologic concentrations, thereby enabling greater maximal efficacy (E_max_) as seen for NNC5840 *in vivo*. Demonstration of this hypothesis would help reconcile the unexpected preclinical efficacy of biased GLP-1R agonists reported here and elsewhere.

Tirzepatide is amongst the best-in-class weight loss therapies despite eliciting partial Gsα agonism at the GLP-1R. The improved efficacy of tirzepatide has been previously ascribed both to its full, potent GIPR agonism profile and to its partial, biased GLP-1R signaling profile [9,[21], [22], [23], [24]]. Interestingly, the broad pattern of weight loss induced by partial, biased GLP-1R agonists (lower potency but higher efficacy) in rodents aligns with that of tirzepatide in the clinic. The published clinical data suggest the intriguing notion that the efficacy of tirzepatide impinges on multiple biologic systems in which GIPR agonism alone can drives weight loss [[21], [22]], improves insulin sensitivity, and suppresses nausea [21,25]. While speculative, it is possible that the latter effect to suppress nausea may facilitate the delivery of tirzepatide at higher tolerable doses than balanced GLP-1R mono-agonists. Our data would predict that the increased efficacy of biased GLP-1R agonism would manifest primarily at such higher doses, helping account for the improved efficacy of tirzepatide. It is noteworthy that recent work by Hinds et al., show a reduction in kaolin intake in rodents treated with a cAMP-biased GLP-1R agonist compared to semaglutide, suggesting this mechanism may result in dampened aversive effects despite greater weight lowering [6].

While conclusions about the clinical effects of biased GLP-1R agonism derived from the tirzepatide data are confounded by its dual GLP-1R/GIPR co-agonist profile, recent human pharmacogenomics data strongly suggest a human translational quality for the superior efficacy of biased GLP-1R agonism seen in rodents. Patients with rare, putative loss of function genetic variations in β-arrestin 1 showed superior HbA1c lowering in response to GLP-1R agonists across a subset of clinical trials [26]. These variants were not directly associated with improved glucose control at baseline but rather display a pharmacogenomic interaction with GLP-1R agonists. The pharmacology exemplified in our work is predicted to confer all patients with the genetic advantage illustrated in this pharmacogenomics association. However, we cannot draw direct conclusions about the weight lowering association of GLP-1R agonists and these rare β-arrestin variants as it was not assessed in the published work. Additionally, numerous small molecule GLP-1R agonists including orforglipron (LY3502970) [27] are heavily cAMP-biased, partial agonists. Orforglipron has shown significant effects to lower body weight in a phase 2 study of patients with obesity, driving weight loss comparable to that of full, unbiased peptide agonists like semaglutide [28]. While a dedicated clinical study comparing a partial-Gsα, cAMP-biased GLP-1R mono-agonist to a full, balanced one is necessary to demonstrate superiority, the preclinical data along with the published human genetics and clinical pharmacology data are strongly suggestive of the possibility.

Our data provide a clear rationale for considering partial agonism, β-arrestin recruitment, and signaling bias quantification in the early drug discovery screening process for GLP-1R agonists. However, obesity pharmacotherapy has quickly evolved to favor unimolecular multireceptor agonists like tirzepatide, survodutide and retatrutide, or loose combinations of distinct pharmacologies like CagriSema. It is not clear whether biased agonism is a key consideration for other receptors of interest in treating metabolic disease. For example, a recent report suggests that β-arrestin recruitment facilitates the insulinotropic actions of GIPR in rodents [29]. Thus, while the partial, biased agonism at GLP-1R may aid efficacy, a full, balanced profile at other receptors may prove preferable. Nevertheless, the phenomenon of biased or selective signaling may serve as a mechanism to optimize candidates at various target receptors [10]. Optimization of multiple parameters on multiple target receptors creates a complex problem to solve with traditional structure–activity relationship (SAR) campaigns, analogous to a Rubik's Cube. However, we postulate that the convergence of resolved ligand:receptor structure data and artificial intelligence trained on large incretin receptor SAR data sets may serve as useful tools for future drug discovery efforts.

Partial-Gsα, cAMP-biased GLP-1R agonists are demonstrated by our data and other reports to confer superior weight lowering efficacy compared to balanced agonists. Crucially, our data indicate that signaling bias significantly correlates to preclinical weight lowering efficacy, whereas the industry standard cAMP accumulation assay used here does not. These data collectively commend a more holistic approach to early drug discovery screening programs that takes at least Gsα/cAMP and β-arrestin signaling into consideration when selecting candidate molecules.

## CRediT authorship contribution statement

**Jonathan D. Douros:** Writing – review & editing, Writing – original draft, Visualization, Validation, Supervision, Project administration, Methodology, Investigation, Formal analysis, Data curation, Conceptualization. **Aaron Novikoff:** Writing – review & editing, Writing – original draft, Visualization, Methodology, Investigation, Formal analysis, Data curation, Conceptualization. **Barent DuBois:** Methodology, Formal analysis, Data curation, Conceptualization. **Rebecca Rohlfs:** Visualization, Validation, Methodology, Investigation, Formal analysis, Data curation, Conceptualization. **Jacek Mokrosinski:** Visualization, Validation, Methodology, Formal analysis, Data curation, Conceptualization. **Wouter F.J. Hogendorf:** Writing – review & editing, Methodology, Conceptualization. **Robert Augustin:** Formal analysis, Data curation, Conceptualization. **Myrte Merkestein:** Writing – original draft, Formal analysis, Data curation, Conceptualization. **Lene Brandt Egaa Martini**: Writing – original draft, Formal analysis, Data curation, Conceptualization. **Lars Linderoth:** Writing – original draft, Supervision, Methodology, Formal analysis, Data curation, Conceptualization. **Elliot Gerrard:** Writing – original draft, Visualization, Methodology, Investigation, Formal analysis, Data curation, Conceptualization. **Janos Tibor Kodra:** Writing – original draft, Formal analysis, Data curation, Conceptualization. **Jenny Norlin:** Writing – original draft, Formal analysis, Data curation, Conceptualization. **Nikolaj Kulahin Roed:** Writing – original draft, Visualization, Project administration, Data curation, Conceptualization. **Anouk Oldenburger:** Writing – original draft, Data curation, Conceptualization. **Stephanie A. Mowery:** Writing – original draft, Visualization, Methodology, Investigation, Formal analysis, Data curation, Conceptualization. **Maria Waldhoer:** Writing – original draft, Visualization, Formal analysis, Data curation, Conceptualization. **Diego Perez-Tilve:** Writing – original draft, Visualization, Supervision, Resources, Data curation, Conceptualization. **Brian Finan:** Writing – original draft, Supervision, Project administration, Conceptualization. **Steffen Reedtz-Runge:** Writing – original draft, Formal analysis, Data curation, Conceptualization. **Timo D. Müller:** Writing – original draft, Supervision, Data curation, Conceptualization. **Patrick J. Knerr:** Writing – review & editing, Visualization, Supervision, Project administration, Investigation, Data curation, Conceptualization, Writing – original draft.

## Declaration of competing interest

JDD, BD, RR, JM, WFJH, RA, MM, LBEM, LL, EG, JTK, JN, NKR, AO, SAM, MW, BF, SR-R, and PJK are or were employees of Novo Nordisk. Novo Nordisk provided funding. BF is a current employee of Eli Lilly & Co. TDM receives research funding from Novo Nordisk and has received speaking fees from Eli Lilly, AstraZeneca, Novo Nordisk and Merck. The remaining authors declare no competing interests.

## Data Availability

The authors do not have permission to share data.
