# Supplementary material for: A GLP-1 analogue optimized for cAMP-biased signaling improves weight loss in obese mice
Source: Mol Metab. 2025 Mar 27;100:102124. doi: 10.1016/j.molmet.2025.102124 (PMC12398856; doi:10.1016/j.molmet.2025.102124)
Supplement: Multimedia component 1 [file mmc1.pdf]

Supplementary Material for:

Douros et al, “A GLP-1 analogue optimized for cAMP-biased signaling improves weight loss in obese mice”

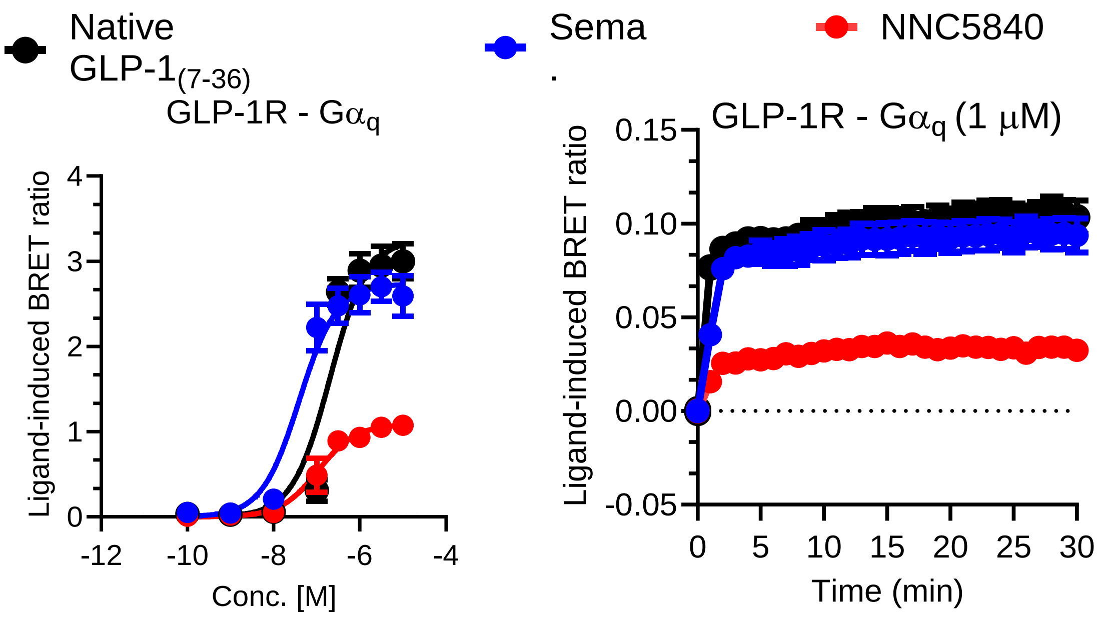

Supplemental Figure 1: Gq signaling by the GLP-1R in response to semaglutide, NNC5840, and native GLP-1.

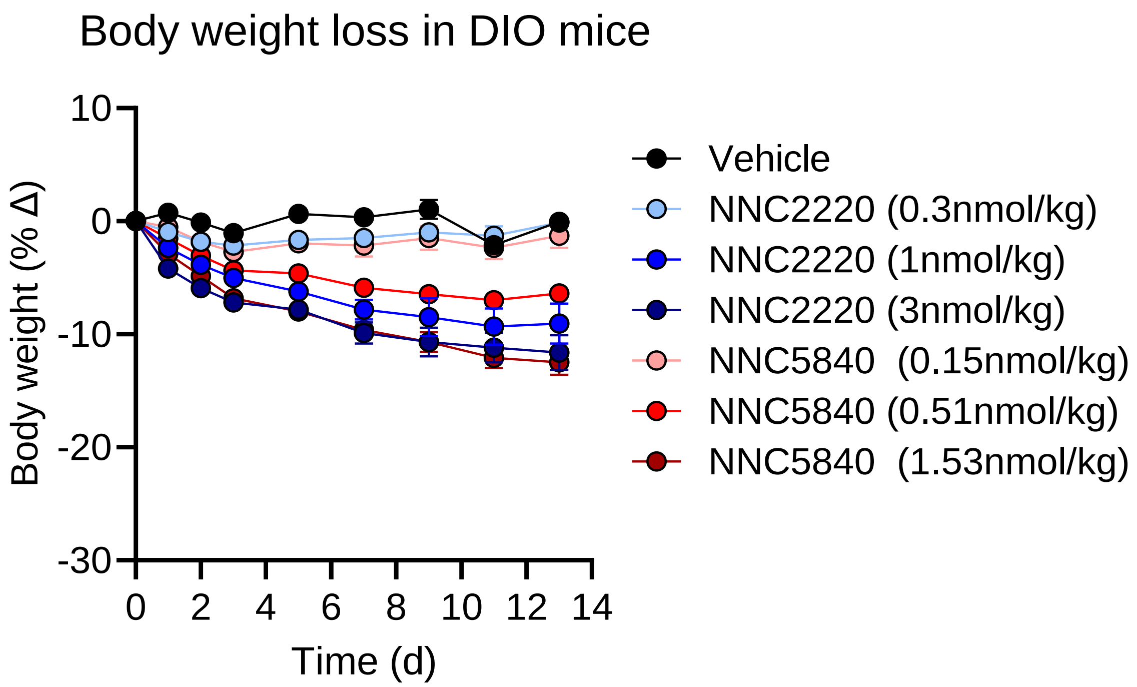

Supplemental Figure 2. <u>NNC5840 induces comparable body weight loss compared to semaglutide in mice at sub-maximally efficacious doses.</u> Body weight loss in DIO mice treated with fixed doses of either semaglutide surrogate (NNC2220; 0.3, 1, or 3 nmol/kg) or NNC5840 (0.15, 0.51, 1.53 nmol/kg).

Supplemental Table 1. *In vitro* GLP-1R potency data generation for Figure 1C, D.

Compound	cAMP E_{max}	cAMP EC_{50} (M)	Barr E_{max}	Barr EC_{50}
Native GLP-1	100	5.9585E-12	100	2.352E-08
Sema	100	1.5712E-12	100	1.323E-08
NNC5840	100	1.92E-11	9.6	2.015E-08
acylEx4-asp3	96	5.01E-10	81	2.51E-08
acylEx4-phe1	97	1.58E-09	14	5.01E-08
NNC5821	100	1.22E-11	23	0.000000331
	Log(data)			
	cAMP E_{max}	cAMP EC_{50}	Barr E_{max}	Barr EC_{50}
Native GLP-1	6.64385619	11.22486306	6.64385619	7.628562683
Sema	6.64385619	11.80376853	6.64385619	7.878440156
NNC5840	6.64385619	10.71669877	3.263034406	7.69572495
acylEx4-asp3	6.584962501	9.3	6.339850003	7.6
acylEx4-phe1	6.599912842	8.8	3.807354922	7.3
NNC5821	6.64385619	10.91221858	4.523561956	6.480172006
	RA cAMP	RA Barr	beta	
Native GLP-1	1	1	1	
Sema	1.0515735	1.032755512	1.0182211	
NNC5840	0.954728687	0.4954596	1.9269557	
acylEx4-asp3	0.821173603	0.950669657	0.8637844	
acylEx4-phe1	0.778788622	0.548382114	1.4201569	
NNC5821	0.972147146	0.578367837	1.6808458	

Supplemental Table 2. Summary pharmacokinetic data for NNC5840 and semaglutide (compound exposure curves in Figure 3C)

Compound	Dose (nmol/kg)	$t_{1/2}$ (h)	C_{max} (nM)	AUC_{last} (h*nmol/L)
Semaglutide	1	N.D.	5	27.2
Semaglutide	3	9.26	16.9	289.23
Semaglutide	5	7.72	30.7	489.39
NNC5840	1	21.71	8	148.3
NNC5840	3	21.06	36.4	656.68
NNC5840	5	29.22	49.4	953.56
